# Notes on Poisoning in the Coal Tar Industries

**Published:** 1918-11

**Authors:** F. G. Möhlau

**Affiliations:** Buffalo N. Y.; Physician in Charge Natl. Aniline & Chem. Co. Buffalo, N. Y.


### Notes on Poisoning in the Coal Tar Industries.

By DR. F. G. MÖHLAU, Buffalo, N. Y.

Physician in Charge Natl. Aniline & Chem. Co. Buffalo, N. Y.

The manufacture of coal tar products in almost endless variety has developed tremendously within the last few years. Since the outbreak of this world's war the coal tar industry has introduced in this country an entirely new set of problems. As the fields of investigation in this line grow larger and more complicated, the needs of workers in the coal tar industries make greater demands upon our ingenuity. These new conditions confront the workers in the manufacture of munitions, dyes, and rubber goods. Physicians who are responsible for the health and welfare of the great army of employees in these various factories are just beginning to meet the demands in a scientific way.

It is only during the last few years that clinical records can be found of such pathological conditions as benzol dermatitis, for example, to mention only one of many conditions resulting from aniline poisoning. It is unfortunate that we have so few observations on record to aid the industrial physician. It seems that the seriousness of these conditions is not sufficiently grasped by the profession at large. It is necessary for every physician to make himself acquainted with the dangers connected with the manufacture of munitions, rubber goods, dyes, and aniline products in general. The various gases evolved in the process of manufacture of munitions and colors have a most deleterious effect upon the blood elements and the circulation as well as upon the genito-urinary tract and liver. The gases evolved in the nitration of various coal tar products, when carelessly inhaled by the worker, have a very disastrous effect on the blood cells where their destructive power is manifested in a most serious manner. The benzol vapors, whether bi-nitro-benzol, or tri-nitro-chlor-benzol act as very strong toxins on the blood cells and their effects on the nerves and nerve centers are severe and of a most alarming character.

In this form of poisoning, the first symptom that strikes us is decided cyanosis of the lips and tongue, sallow color of the face, apathetic expression and a varying degree of blueness of the finger nails as in poisoning by acetanilid. As a rule, the breathing is difficult and shallow, often amounting to dyspnoea.

We find that when a man has been living for any length of time in an atmosphere charged with the fumes of bi-nitro-benzol or diamin, he will develop the following symptoms:

Anaemia; marked gastro-intestinal disturbance; congestion of the vasculo-glandular network of the liver, resulting in congestion and derangement of function; enlargement of the spleen; jaundice in varying degree; constipation is marked; diarrhoea, only in summer on cloudy and oppressive hot days. The temperature is usually reduced to subnormal. I have very rarely found a rise in temperature. Conjunctivitis is usually pronounced and recurs frequently. The patient feels irritable and depressed. The urine: specific gravity high; highly colored with bile pigment; phosphates excessive in amount; albumin absent in early stages, unless kidneys have already suffered structural changes, or hematuria has set in. The pulse is full, soft, and compressible, with complete loss of tone; rapid at first, then slower after a short time. The respiration is shallow and difficult. After a short period, the patient will complain of muscular pains in the legs and arms resembling rheumatism. Intercostal pains are frequently observed. There is a short, dry cough with little tenacious mucus; subacute bronchitis is not infrequent.

Our treatment has been very satisfactory when regularly carried out under close observation. Lactic acid has been found most effective in the form of buttermilk and whey, or milk in large quantity for diuresis. After the surroundings have been changed, affording an unlimited supply of fresh, moist air we attend to the activity of the skin. Cool sponges followed by friction are of marked benefit. Later the hot bath and Turkish baths are a necessity. Following an acute attack of this poisoning, a period of rest and recreation becomes necessary. The food question must not be overlooked. Not resisting power not immunity can be established,—only improving the general health. A man once poisoned must not be allowed to be exposed to the same surroundings.

During the four years I have been called upon to watch over the health and welfare of from 500 workmen in 1915 to 2000 and more in 1917, I have had the opportunity of treating about 641 cases of aniline poisoning in various stages of severity. I am glad and proud to say not a single death has occurred among all these victims of poisoning. I may possibly account for this by the fact that whenever I observe a worker even slightly cyanosed I insist on his taking rest; the more severe cases are immediately sent to the hospital which they are permitted to leave, as a rule, whenever the cyanosis has subsided and the hemoglobin is beginning to mend. And yet, leaving the hospital does not mean a return to work or a relaxation of treatment. The patient is to live in fresh air under hygienic conditions, with well regulated diet and proper medication. A hot bath once daily is considered essential with massage and a cool sponge in the morning. I

recommend milk in all forms, especially buttermilk and sour milk which certainly prove the value of the Bacillus Bulgaricus. Nux vomica has no superior as a remedy. I give 8 to 10 minims three or four times daily and it never fails to produce a result. Blaud's mass with manganese and arsenic is also given four times a day in full doses with excellent results. Oxygen is administered freely at frequent intervals as long as cyanosis exists. Massage is kept up. Cathartics are given as long as an excess of bile is found in the urine or while the serum is bile colored. As long as the liver shows enlargement and the spleen remains congested, a dose of ergot is given with good results. One teaspoonful twice or three times a day. In the aniline industry we have to guard against sequalae of acute attacks of poison by inhalation of gases given up during the nitrating process, be it absorption by skin or ingestion by mouth or inhalation of finished color product, the effects on bladder, ureter and kidney are of such a character as to puzzle the average physician. The effects produced in the bladder, I have been able to observe repeatedly but on account of lack of time, I have been unable to give closer study to the action on the kidneys. I have seen several bladder cases that present typical pictures of cauliflower growth, where no malignancy could be demonstrated. Cystascopically the picture presented is a growth mostly like a red coral or cauliflower. In one case the bladder was almost obliterated by such a growth. The least local irritation produced profound hemorrhage with very little or no pain, the anemia produced hurrying the fatal exit. I had the opportunity to observe four such cases in the late stages, rendering surgical interference impossible and any medical treatment was absolutely of no avail. Due to pressure of other work, I have been unable to follow these conditions from a pathological standpoint.

237 East Utica St., Buffalo, N. Y.

---

**Tuberculous Meningitis.** Tilli, Giornale de Med. Chir., March, recommends injecting 1-3 c.c. of the patient's spinal fluid on alternate days. He reports a case apparently cured for three years, one arrested for the same time though with persistence of blindness and hydrocephalus, one with a remission of 20 months followed by recedive and death.

---

**Typhoid Maladies of the Horse.** R. Combes, Le Prog. Med., Apr. 6, has found a bacillus closely related to B. paratyphosis B., infectious not only to horses but to man, by accidental conveyance or use of improperly cooked meat. Veterinarians and hostlers have noted cases similar to human typhoid in horses.

